# Crosstalk Between Senescent Bone Cells and the Bone Tissue Microenvironment Influences Bone Fragility During Chronological Age and in Diabetes

**DOI:** 10.3389/fphys.2022.812157

**Published:** 2022-03-21

**Authors:** Thibault Teissier, Vladislav Temkin, Rivka Dresner Pollak, Lynne S. Cox

**Affiliations:** ^1^Department of Biochemistry, University of Oxford, Oxford, United Kingdom; ^2^Division of Medicine, Department of Endocrinology and Metabolism, The Hadassah Medical Center, Faculty of Medicine, The Hebrew University of Jerusalem, Jerusalem, Israel

**Keywords:** aging, senescence, bone fragility, diabetes, RAGE, glycation, senolytic, mTOR

## Abstract

Bone is a complex organ serving roles in skeletal support and movement, and is a source of blood cells including adaptive and innate immune cells. Structural and functional integrity is maintained through a balance between bone synthesis and bone degradation, dependent in part on mechanical loading but also on signaling and influences of the tissue microenvironment. Bone structure and the extracellular bone milieu change with age, predisposing to osteoporosis and increased fracture risk, and this is exacerbated in patients with diabetes. Such changes can include loss of bone mineral density, deterioration in micro-architecture, as well as decreased bone flexibility, through alteration of proteinaceous bone support structures, and accumulation of senescent cells. Senescence is a state of proliferation arrest accompanied by marked morphological and metabolic changes. It is driven by cellular stress and serves an important acute tumor suppressive mechanism when followed by immune-mediated senescent cell clearance. However, aging and pathological conditions including diabetes are associated with accumulation of senescent cells that generate a pro-inflammatory and tissue-destructive secretome (the SASP). The SASP impinges on the tissue microenvironment with detrimental local and systemic consequences; senescent cells are thought to contribute to the multimorbidity associated with advanced chronological age. Here, we assess factors that promote bone fragility, in the context both of chronological aging and accelerated aging in progeroid syndromes and in diabetes, including senescence-dependent alterations in the bone tissue microenvironment, and glycation changes to the tissue microenvironment that stimulate RAGE signaling, a process that is accelerated in diabetic patients. Finally, we discuss therapeutic interventions targeting RAGE signaling and cell senescence that show promise in improving bone health in older people and those living with diabetes.

## Bone Integrity Is Reliant on Intercellular Crosstalk and Interactions With the Bone Microenvironment

**FIGURE 1:**
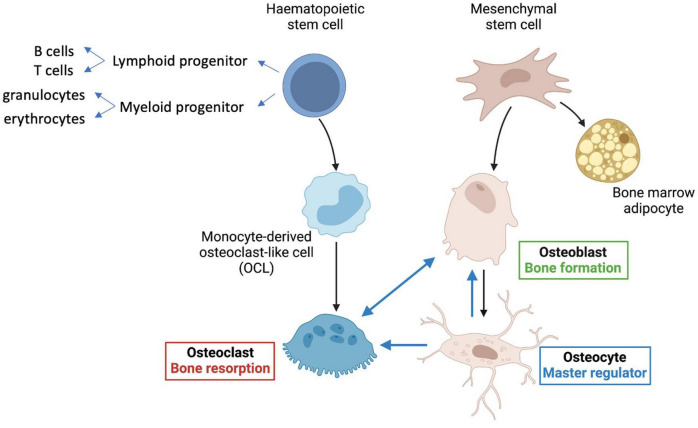
Key cell types in bone. Hematopoietic stem cells (HSCs) give rise to lymphoid progenitors that lead to formation of B and T cells. Myeloid progenitor cells give rise to all classes of granulocytes, monocytes, and red cell precursors (erythroblasts), as well as osteoclasts, *via* monocyte derived osteoclast-like cells (OCLs). Mesenchymal stem cells (MSCs) predominantly give rise to bone-forming osteoblasts, osteocytes, and also bone marrow adipocytes. The longest-lived cells in bone are the osteocytes, which regulate formation of both osteoclasts and osteoblasts and are major contributors to mineralization (see text for details).

Bone is a highly complex organ, comprising multiple different cell types as well as nerves and blood vessels within an inorganic calcium phosphate matrix supported by collagen and other scaffold proteins. Hematopoietic stem cells (HSCs) within bone give rise to red and white blood cell lineages, as well as platelets and osteoclasts, which are multinucleated cells derived from the monocyte-macrophage lineage of hematopoietic origin (Figure 1). Osteoclasts are formed by cell fusion events and so are large and multinucleate—they are responsible for bone resorption important in bone remodeling. Bone marrow mesenchymal stem cells (MSCs, also known as BM-MSCs), are precursors of lining cells, marrow adipocytes and bone-forming osteoblasts. Osteocytes are long-lived bone cells derived from osteoblasts (Figure 1), and act as master-regulators co-ordinating inputs from mechanical loading, microenvironmental cues (e.g., paracrine and inflammatory signaling) and hormonal signals.

Crosstalk between bone cell types, as well as *via* influences from the bone microenvironment, ensure tight regulation and allow dynamic responses to environmental cues to regulate the balance between bone formation and bone resorption. Certain pathological states, including malnutrition, older age, chronic inflammation and diabetes can lead to dysregulation of this balance, altering not only formation/resorption directly, but also driving changes in cell fates upon stem cell proliferation: together these can predispose to bone fragility.

## Rankl Signaling Regulates Bone Cell Differentiation

**FIGURE 2:**
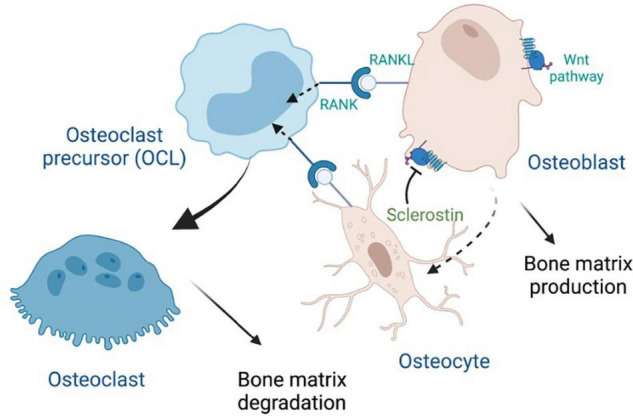
Crosstalk between major bone cells through RANK, RANKL and Wnt signaling, with inhibition by sclerostin. Osteoprotegerin (OPG), which is secreted by osteoblasts, can also bind to RANKL (not shown).

Osteocytes are the most abundant and longest-lived cells in bone; they serve as mechanosensors (*via* dendritic networks), and orchestrate formation and resorption of bone in part through production of RANKL (Receptor activator of nuclear factor κ B ligand), a transmembrane protein of the TNF superfamily (Kong et al., 1999) in adult bone to mediate osteoclastogenesis (Nakashima et al., 2011; Xiong et al., 2011). RANKL binds to RANK, a transmembrane protein that is expressed in the osteoclastic lineage (Figure 2), in forward signaling that drives osteoclast differentiation. It has been recently discovered that marrow adipogenic lineage precursors (MALPs) also express RANKL and interact with cells of the monocyte-macrophage lineage in the bone marrow niche to promote osteoclastogenesis (Yu et al., 2021). Osteoclasts influence osteoblast differentiation by reverse signaling of RANKL (Ikebuchi et al., 2018) and forward signaling *via* Ephrin2/EphB4, whereby osteoclast-secreted ephrinB2 binding to the receptor EphB4 enhances osteogenic differentiation (Zhao et al., 2006). Reverse signaling is mediated *via* osteoclast secretion of vesicles containing RANK that binds to osteoblastic RANKL and induces the expression of the osteoblastic transcription factor RUNX2 (Figure 2). Notably, RANKL is also made by osteoblasts that produce osteoprotegerin (OPG), a decoy receptor of RANKL and negative regulator of RANKL forward signaling (Boyce and Xing, 2008). WNT signaling in osteoblasts drives accumulation of β-catenin, which in turns leads to osteoblastogenesis. Notably, osteocytes produce sclerostin, an inhibitor the WNT signaling pathway in osteoblasts (Figure 2), thereby regulating osteoblast generation and function.

## Bone Fragility in People Living With Diabetes

Type 1 (T1D) and type 2 diabetes (T2D) are major risk factors for premature development of age-related diseases including cardiovascular disease, cognitive impairment, renal dysfunction, sarcopenia, and skeletal fragility. Investigating the role of aging mechanisms in diabetes holds promise for identifying new pathways and targets to alleviate diabetic complications that are not fully overcome by currently available anti-diabetic therapies.

Skeletal fragility is a newly recognized serious complication of both T1D and T2D leading to significantly increased risk of sustaining a fracture after minimal trauma. In addition, impaired fracture healing and prolonged hospitalization post-fracture lead to loss of functional capacity and independence, as well as increased post fracture mortality (Ferrari et al., 2018). While fracture risk is increased in both T1D and T2D, hip fracture risk is dramatically higher (3–6-fold) in T1D compared to non-diabetics and increases with age (Ferrari et al., 2018). Patients with T2D are also at an increased risk of sustaining a hip fracture (1.4–1.7-fold), vertebral fracture (1.3–2-fold) and upper arm and ankle fracture (1.5-fold) compared to non-diabetics (Janghorbani et al., 2007; Melton et al., 2008; Khosla et al., 2021). Risk factors for skeletal fragility in diabetes include disease duration (Majumdar et al., 2016), poor glycemic control (Li et al., 2015; Farr and Khosla, 2016; Khosla et al., 2021), insulin therapy (Losada-Grande et al., 2017), and micro-vascular complications (Shanbhogue et al., 2017).

The aetiology of skeletal fragility in T1D and T2D is multi-factorial (Napoli et al., 2017). Bone mass, as evaluated by bone mineral density (BMD), is only modestly reduced in adult T1D patients compared to age- and sex- matched controls (Napoli et al., 2017), and does not fully explain the high fracture risk. Moreover, BMD in T2D is higher or normal compared to age- and sex-matched controls potentially because higher body mass imposes greater mechanical loading (Bilha et al., 2021), however, fracture risk is significantly higher. Alterations in bone architecture and reduced bone quality that are not captured by BMD have been implicated in the pathogenesis of reduced bone mechanical strength in T1D and T2D. These alterations include decreased cortical bone thickness (Samelson et al., 2018), increased cortical bone porosity, fewer and thinner trabeculae in trabecular bone, changes in bone material properties and lower toughness, as well as compositional changes including increased advanced glycation end-products (AGEs), and higher non-enzymatic cross-links increasing the propensity to bone fragility (Hunt et al., 2019; Khosla et al., 2021).

At the tissue level, T1D and T2D are both characterized by low bone formation (Hygum et al., 2017) that can result in inadequate repair of micro-damage and the accumulation of aging bone. Increased bone resorption has been reported in some studies (Napoli et al., 2017). While bone histology data in human diabetic patients is limited, studies of serum bone turnover markers (BTMs) in diabetic patients consistently show reduced levels of markers of bone formation, procollagen type 1 amino-terminal propetide (P1NP) and osteocalcin, while serum sclerostin, an inhibitor of bone formation *via* the canonical WNT pathway (Figure 2), is elevated (Piccoli et al., 2020). Additional alterations at the tissue level that have been implicated as contributors to diabetic bone fragility include increased bone marrow adiposity and evidence of inflammation in the bone marrow microenvironment (Napoli et al., 2017) (we discuss sources of this inflammation further below). At the cellular level, multiple cell types within the bone micro-environment including bone mesenchymal stem cells, osteoblasts, lining cells, osteocytes, marrow adipocytes, and osteoclasts display abnormal phenotypes in diabetes (Napoli et al., 2017).

The osteocyte appears to play a central role in diabetes-induced bone deterioration; decreased sensitivity of osteocytes to mechanical load under conditions of hyperglycemia has been demonstrated both *in vitro* and *in vivo*. In an osteocyte cell line model (MLO-Y4), high glucose impaired the calcium signaling response to fluid flow stimulation (Tanaka et al., 2015a), while *in vivo*, a diminished anabolic response to loading was demonstrated in heterozygous C57BL/6-Ins2Akita/J (Akita) male mice, an animal model of T1D (Hie et al., 2011). In humans, elevated serum sclerostin, a product of the osteocyte, is found in both T1D and T2D patients (Napoli et al., 2018; Piccoli et al., 2020).

## Non-Enzymatic Glycation Increases With Age and Leads to Tissue Damage

Long-term dysregulation of plasma glucose levels in patients with diabetes can accelerate formation of advanced glycation end-products (AGEs). AGEs are a family of molecules resulting from the non-enzymatic and irreversible interaction between carbonyl compounds, such as glucose or other sugars, and nucleophiles, such as proteins and lipids, which greatly modifies their structure, and thereby alters their functions (Chaudhuri et al., 2018).

It is now known that AGEs are formed not only from glucose, but also from glucose-derived dicarbonyl precursors that are much more reactive and prone to form AGEs than glucose itself. Therefore, studies using proteins glycated *in vitro* with glucose do not necessarily recapitulate the variety, nor the quantity, of AGEs naturally found *in vivo* under physiological or diabetic conditions. It is thought that intra-cellular hyperglycemia may be a driver of both intracellular and extracellular AGEs (Shinohara et al., 1998). Three main dicarbonyl precursors have been identified: (i) glyoxal arising from glucose oxidation, (ii) 3-deoxyglucosone, and (iii) methylglyoxal, which has a key role in production of reactive AGEs (Thornalley et al., 2003). As well as the more commonly described protein adducts, AGE-modified DNA has also been reported in diabetic patients (Li et al., 2006), while glyoxal leads to oxidative damage including elevated levels of 8-oxo-G lesions in DNA; telomeres at the end of chromosomes are G-rich and especially susceptible to this type of damage. Measurements of AGEs *in vivo* in tissues and serum in diabetes usually assess common glycation products such as HbA1c (glycated hemoglobin), 1-carboxymethyl-L-lysine (CML), and/or pentosidine, a fluorescent AGE cross-link formed between lysine and arginine. Through these measures, AGEs are found to greatly increase with age in tissues, and their rate of accumulation is inversely associated with longevity in several species, including humans (Sell et al., 1996, 2000; Semba et al., 2009). As well as correlating with older age, AGEs may be directly responsible for tissue aging, as demonstrated by studies on cataracts (Monnier and Cerami, 1981; Dunn et al., 1989; Ahmed et al., 1997; Tessier et al., 1999).

Beyond aging and diabetes, AGEs are also produced in various tissues under conditions of eating high-fat, high sugar processed foods rich in saturated fatty acids, cigarette smoking, chronic alcohol consumption and inflammation (Asadipooya and Uy, 2019) notably these conditions are also associated with poor health outcomes.

## AGEs Negatively Impact on Bone Cells and Tissues

Tissues containing long-lived proteins and with low turnover, such as bone, are particularly vulnerable to glycation modifications. AGEs adversely affect bone cells and bone mechanical properties. In contrast to the mineral phase of the bone that is not affected by glycation (Vashishth et al., 2001; Tang et al., 2007), non-enzymatic AGE cross-links within type 1 collagen, the main protein in bone, result in increased bone stiffness, reduced bone flexibility and altered material and biomechanical properties (Napoli et al., 2017). *Ex vivo* glycation of cortical bone samples also led to the formation of large numbers of crosslinks, notably pentosidine, within the collagen network, leading to increased stiffness, which greatly altered the mechanical properties of the bone (Vashishth et al., 2001; Willett et al., 2013). AGEs, notably pentosidine, have been found to accumulate with age in human bones, especially in the cortical bone where an exponential accumulation has been shown (Odetti et al., 2005); both pentosidine and collagen crosslinking are associated with decreased bone quality and impaired loading capacity (Neumann et al., 2014).

There is a strong correlation between levels of AGEs and bone fragility, as observed in a large cohort of old participants, where higher CML levels at baseline were associated risk of hip fracture over a 9 year-follow-up (Barzilay et al., 2014). Overall, the level of AGEs, rise with age, as does collagen-crosslinking, and this reduces bone quality and strength.

Glycation products, either within insoluble matrices or in soluble proteins, have been shown to inhibit the resorption function of osteoclasts *in vitro*, and glycated matrices from bones were also processed less efficiently (Valcourt et al., 2007; Li et al., 2016). AGEs are reported to inhibit monocyte differentiation into osteoclasts (Tanaka et al., 2019), and markedly impact on the activity of early osteoclast-like cells (OCLs) until the fusion stage, though AGEs did not inhibit resorption by mature osteoclasts and even led to increased podosome number in fully differentiated osteoclasts (Valcourt et al., 2007; Li et al., 2016). Mechanistically, AGEs may directly interfere with osteoclast differentiation through RANKL (RANK ligand) signaling, since glycolaldehyde-modified BSA was shown to repress expression of RANK and TRAF6 (TNF receptor-associated factor 6), preventing maturation of pre-osteoclasts (Tanaka et al., 2019).

Osteoblasts are also markedly affected by AGEs, leading to reduced differentiation, proliferation, and viability *in vitro* and decreased expression of collagen type I, osteocalcin, osterix, osteopontin, RUNX2, BMP-2 (bone morphogenetic protein 2) and alkaline phosphatase, while inflammatory pathways are promoted (Franke et al., 2011; Okazaki et al., 2012; Cheng et al., 2018). The alteration of the osteogenic potential of human periodontal ligament stem cells by AGEs seemed to be dependent of PKCβ2 phosphorylation, while GLP-1 (glucagon-like peptide-1) exposure prevented most of these effects (Wang et al., 2020). In rat bone marrow mesenchymal stem cells (BM-MSCs), AGEs mainly impaired osteogenesis via down-regulation of the peroxisome proliferator-activated receptor γ (PPAR γ) (Wang et al., 2021). Notably, glucose alone had none of these effects (Okazaki et al., 2012), though a combination of glucose and AGEs appears to diminish mineralization by MC3T3-E1 cells (Ogawa et al., 2007). Patient-derived osteoblasts from older adults showed increased RANKL levels upon AGE exposure accompanied by increased inflammatory signaling (Franke et al., 2011). While AGEs are generally considered to form in the extracellular matrix or on the cell surface, intracellular AGEs have also been shown to accumulate within cultured mouse osteoblastic cells, leading to ER stress and apoptosis (Suzuki et al., 2020). If recapitulated *in vivo*, this finding could provide a partial explanation for age-related osteoporosis through loss of osteoblasts. Moreover, osteocytes are also impacted by AGEs, with increased expression of sclerostin (which inhibits the Wnt pathway) observed after exposure of osteocytelike MLOY4 cells to AGEs, although whether AGEs increase or decrease RANKL expression in osteocytes is less clear as reports are inconsistent (Tanaka et al., 2015a; Notsu et al., 2017; Zhang et al., 2019).

## Advanced Glycation End-Products Are Pathogenic in Diabetic Bone

As diabetes is primarily characterized by dysregulated glucose levels, and AGEs form as a result of glycation, it is not surprising that AGEs are elevated in diabetic patients—indeed glycated hemoglobin (HbA1c) is routinely used to monitor blood sugar control. AGEs actively contribute to a number of diabetic complications such as nephropathies, neuropathies, retinopathies and cardiomyopathies (Brownlee, 2001; Singh et al., 2014; Chaudhuri et al., 2018). Below we consider the impact of AGEs on bone in people living with diabetes.

Advanced glycation end-products contribute to diabetes-induced deterioration in bone biomechanical strength. *In vitro*, the administration of synthetic AGE and 25 mM glucose to marrow-derived macrophages and MCT3T3-E1 cells resulted in decreased TRAP-positive multinucleated cell formation and alkaline phosphatase activity, suggesting that AGEs in the presence of hyperglycemia suppress both osteoclast and osteoblast differentiation and function (Park et al., 2021a).

Pre-clinical studies in animal models of diabetes have demonstrated AGEs accumulation in bone (Rubin et al., 2016; Acevedo et al., 2018). In diabetic male mice fed on a high fat diet, increased levels of total fAGEs, pentosidine and carboxymethyl-lysine (CML) were found in bone, while *ex vivo* treatment of femurs obtained from these mice with phenacyl thiazolium chloride (PTC) for *in vitro* removal of glycation products increased bone toughness, a parameter of mechanical strength (LLabre et al., 2021). Glycation of the trabecular bone similarly affects its mechanical properties, notably the damage fraction, post-yield strain energy and energy dissipation, suggesting a higher susceptibility to brittle fracture (Tang et al., 2007). There is a strong correlation between levels of AGEs and bone fragility, as observed in a large cohort of old participants, where higher CML levels at baseline were associated risk of hip fracture over a 9 year-follow-up, even when adjusted for BMD or diabetes, (Barzilay et al., 2014). Overall, the level of AGEs, particularly pentosidine, rises with age, as does collagen-crosslinking, and this reduces bone quality and strength.

As human bone tissue from subjects with T2DM is not readily accessible, surrogate markers of bone AGEs were investigated. Increased urinary excretion of pentosidine was reported to be associated with increased fracture risk in diabetic older subjects (Schwartz et al., 2009). Serum carboxy-methyl-lysine (CML) was shown to be higher in T2DM compared to non-diabetic subjects and associated with incident clinical fracture in elderly diabetics (Dhaliwal et al., 2021). Increased AGEs in skin in post-menopausal women with T2DM was demonstrated by a non-invasive measure of skin autofluorescence (SAF) and was found to be inversely correlated with bone material strength index determined by microindentation (Furst et al., 2016). As both skin and bone are rich in type 1 collagen, skin SAF, is considered a surrogate marker of bone AGEs, in particular pentosidine. Consistent with this, elevated SAF inversely correlated with spine and hip BMD was recently reported in T2DM patients (Yavuz and Apaydin, 2021).

Data on accumulation of AGEs in diabetic bone in humans is limited. In one study where both serum AGEs (pentosidine and total AGEs) and proximal femur AGEs were quantified, no difference in serum AGEs between diabetics and non-diabetics was found, whereas in cortical bone the level of AGEs was slightly higher in diabetics vs. non-diabetics (Karim et al., 2018). In men undergoing hip arthroplasty, increased pentosidine and sugars bound to collagen were found in T2DM vs. non-diabetic subjects (Hunt et al., 2019). In postmenopausal women undergoing hip replacement an increased was detected in the femoral head in patients with T2DM compared with non-diabetic women (Piccoli et al., 2020). The limitation of these studies is the inclusion of diabetic patients undergoing elective arthroplasty for osteoarthritis, since the presence of arthritis may affect the level of AGEs in bone. Analysis of thoracic vertebrae and femurs in a case-controlled study in organ donors during autopsy revealed increased CML in the femoral neck in diabetic vs. non-diabetic subjects (Wolfel et al., 2020). Importantly, in a recently published study, impaired bone composition and material properties were documented for the first time in the hip tissue in T2DM patients who sustained a fragility fracture of the hip (Sihota et al., 2021). The changes observed in the diabetic group included lower mineral:matrix ratio, wider mineral crystals, higher total fAGEs, higher non-enzymatic cross-link ratio (NE-xLR), and altered amide bands. Moreover, there was a strong inverse correlation between NE-xLR and fAGEs and parameters of bone biomechanical strength including post yield strain and energy, and toughness (Sihota et al., 2021).

## Advanced Glycation End-Products Act as Ligands for Pro-Inflammatory Transmembrane Receptor, Rage

Advanced glycation end-products act directly by modifying macromolecular and tissue properties, but also indirectly by interacting with receptors, notably with the receptor for AGEs (RAGE), a transmembrane multi-ligand pattern recognition receptor. RAGE signaling is implicated in numerous pathologies such as inflammatory bowel disease (Wang et al., 2014; Ciccocioppo et al., 2019), and cancer (Sparvero et al., 2009; Leclerc and Vetter, 2015). RAGE signaling triggers a predominantly pro-inflammatory response upon activation by its ligands (Teissier and Boulanger, 2019), as well as generating reactive oxygen species (ROS). Notably, genetic ablation of RAGE in mice (i.e., *rage*^–/–^ genotype) leads to improved kidney morphology and function in later life compared with WT controls (Teissier et al., 2019), potentially because of reduced inflammatory signaling.

RAGE is also implicated in bone pathology in diabetes (Forbes et al., 2003; Misur et al., 2004; Kang et al., 2012), though there is an apparent paradox in the role of RAGE and AGEs in osteoclast formation and function. On the one hand, RAGE has been shown to promote osteoclastogenesis (Ding et al., 2006; Zhou et al., 2006; Hamada et al., 2010)—RAGE ligands, such as S100A8, S100A9, S100A12, or HMGB1 promote RANKL expression in osteocytes and facilitate osteoclast maturation in a RAGE-dependent manner, suggesting that RAGE signaling promotes osteoclast formation (Tanaka et al., 2015b). By contrast AGEs, which act as RAGE’s main activating ligands, appear to inhibit osteoclastogenesis (Valcourt et al., 2007; Li et al., 2016; Tanaka et al., 2019; Park et al., 2021a). This is of particular importance in the case of some aggressive cancers which secrete HMGB1, ultimately leading to bone resorption (Sakamoto et al., 2020). By contrast AGEs, which act as RAGE’s main activating ligands, appear to inhibit osteoclastogenesis (Valcourt et al., 2007; Li et al., 2016; Tanaka et al., 2019; Park et al., 2021a). Therefore, AGE signalling in osteoclasts may involve different signalling downstream of RAGE activation and also RAGE-independent signalling. Indeed, it has been shown that while AGEs can induce inflammation and M1 polarization in bone-marrow derived macrophages (BMDMs), they also impair PPAR-γ expression and signalling (Wang et al., 2021). This down-regulation of PPAR-γ by AGEs was also found in chondrocytes, and was mostly dependent on RAGE and Toll-like receptor 4 (TLR4) signalling (Chen et al., 2013; Ma et al., 2015). This is important since PPAR-γ plays an essential role in osteoclastogenesis: mice in which PPAR-γ was selectively ablated in osteoclasts (using CRISPR/Cas9) showed osteopetrosis i.e. an abnormal increases in bone mass (Wan et al., 2007), while PPAR-γ promotes osteoclast differentiation through up-regulation of c-fos, a key regulator of RANK/RANKL signalling (Wan et al., 2007). Down-regulation of PPAR-γ by AGEs thus directly impairs osteoclast differentiation, while upregulation of PPAR-γ levels by adrenomedullin 2 has been shown to largely restore osteogenesis impaired *in vitro* by AGEs and *in vivo* by diabetes in rats (Wang et al., 2021). Taken together, these findings suggest that RAGE proinflammatory signalling may promote osteoclast differentiation while AGEs inhibit osteoclastogenesis by dampening PPAR-γ signalling.

In addition to transmembrane RAGE, alternative splicing or cleavage of the extracellular domain can give rise to soluble RAGE (sRAGE) which can be measured in the circulation. While sRAGE has been suggested to act as a “decoy,” binding to AGEs and thus decreasing transmembrane RAGE activation, it is notable that a large cohort study has demonstrated an association between sRAGE and frailty (Butcher et al., 2019) and sRAGE has been causally linked to impaired glucose metabolism in Chinese patients with primary hypertension (Wang et al., 2018), though no association was seen with insulin resistance in Bangladeshi patients with type 2 diabetes (Biswas et al., 2015). In the context of bone aging, sRAGE was found to positively associate with markers of bone turnover (osteocalcin and P1NP) in older men, and this association was more apparent in men with diabetes (Lamb et al., 2018). High sRAGE may simply indicate elevated levels of full-length RAGE, from which sRAGE is derived; alternatively, elevated sRAGE may be an adaptation to counteract the pathological effects of high levels of AGEs, or it may itself drive pathology through a currently unknown mechanism. Whatever its role, sRAGE may prove useful as a proxy readout of either glycation levels and/or RAGE inflammatory signaling.

## Rage Influences Bone Structure and Function

A role for RAGE signaling in the pathogenesis of diabetic complications including diabetic nephropathy and diabetic atherosclerosis has been previously reported (Park et al., 1998; Tanji et al., 2000). RAGE also appears to have a very important role in bone metabolism and mechanical properties, with animal studies suggesting that osteoclasts are the cell type most affected by RAGE expression. Young mice genetically null for RAGE (*rage^–^/^–^*) showed an increased bone mass at the femur and greater biomechanical strength, together with a decreased number of osteoclasts (but a similar number of osteoblasts), when compared with age- and sex-matched WT mice with similar levels of blood calcium, phosphate, insulin and glucose (Ding et al., 2006; Zhou et al., 2006; Hamada et al., 2010). The trabecular bone volume was found to be increased in the femur (Ding et al., 2006) but not in the tibia (Hamada et al., 2010) of young *rage^–^/^–^* mice (3–5 months old) while in very young mice (4 weeks old), the trabecular volume was increased in both the femur and tibia (Ding et al., 2006; Zhou et al., 2006), suggesting that RAGE influences the rate of early post-natal bone development. Interestingly, serum IL-6 and pyridinoline (a bone degradation marker) were both decreased in *rage^–^/^–^* mice, suggesting a decreased inflammation profile associated with decreased osteoclast activity (Ding et al., 2006; Zhou et al., 2006). Knock-out of RAGE in mice also improved recovery from induced bone fracture, with faster recovery of bone mineral density, trabecular bone volume and thickness, and decreased synovitis (Seol et al., 2018). Whether RAGE impacts on bone fragility in diabetes is more moot, since in young mice with streptozotocin-induced diabetes, RAGE knock-out did not protect against bone loss, as measured by histomorphometric analyses (Hamada et al., 2010). However, this finding does not rule out a potential effect of RAGE in older diabetic mice.

RAGE has other ligands beyond AGEs including pro-inflammatory proteins that may also play a role in the bone micro-environment in diabetes (Yan et al., 2008; Plotkin et al., 2019), most notably amyloid (Yan et al., 1996; Deane et al., 2012). In a murine model of Alzheimer’s disease (Tg2576), aggregation of amyloid peptide precursors had a biphasic effect—increased osteoclast number and activity, and concomitant decreased trabecular volume was observed in young mice, while the opposite was seen in mice older than 6 months (Cui et al., 2011). *In vitro* results demonstrated that RAGE was involved, as polymerized Aβ accelerated osteoclastogenesis of bone marrow macrophages (BMMs) in WT but not in *rage^–^/^–^* animals. However, soluble RAGE (sRAGE), which increases more rapidly than RAGE with age in this model, was able to block Aβ/RAGE-dependent osteoclastogenesis, through its inhibition of RANKL signaling, thus explaining the difference in bone effects observed between young and old mice (Cui et al., 2011). Another mechanism by which signaling *via* RAGE induces osteoclastogenesis is through the interaction between advanced oxidation protein products (AOPPs) that accumulate with aging (Maciejczyk et al., 2019) and in diabetes (Kalousova et al., 2002), and RAGE in primary bone marrow monocytes. Binding of AOPPs to RANK and RAGE was shown to activate nicotinamide adenine dinucleotide phosphate (NADPH) oxidase, resulting in the generation of reactive oxygen species, phosphorylation of mitogen-activated protein kinases and c-fos, upregulation of the nuclear factor of activated T cell c1 (NFATC1), which *in vitro* promotes differentiation of bone marrow monocytes into mature osteoclasts. *In vivo*, chronic exposure to AOPPs enhanced osteoclastogenesis and bone loss in mice, which could be alleviated by the administration of the NADPH oxidase inhibitor apocynin (Zhuang et al., 2021). Hence AOPPs act as additional ligands for RAGE, with effects on bone.

RAGE signaling may play a role in a number of bone cell types in addition to osteoclasts. It is expressed in osteoblasts, with high levels observed in fully differentiated osteoblasts (McCarthy et al., 1999). Overexpression of RAGE in the osteoblast cell line MC3T3-E1 led to decreased proliferation, partly through inhibition of Wnt signaling, and subsequent diminished PI3K phosphorylation (Li et al., 2012), consistent with reduced osteoblastogenesis observed in the long bones of aging mice (Rauner et al., 2008). RAGE is also implicated in inflammatory signaling by osteocytes, since exposure of osteocyte-like MLOY4 cells to AGEs stimulated production of IL-6 and VEGF-A (vascular endothelial growth factor A), while FPS-ZM1, a RAGE antagonist, prevented this effect, potentially by limiting the activation of ERK, STAT3, and p38 signaling (Chen et al., 2017). In a cross-sectional human study, the osteogenic differentiation potential of peripheral blood mononuclear cells was markedly reduced (by ∼12-fold) in patients with type 2 diabetes compared with healthy controls, accompanied by high levels of apoptotic markers, and greatly elevated cellular RAGE:sRAGE ratios (Phimphilai et al., 2017).

Bone marrow mesenchymal stem cells (BM-MSCs) are also adversely affected by AGEs present in diabetic conditions, both *in vitro* and *in vivo*. Glyceraldehyde and glycolaldehyde-derived AGEs inhibited proliferation and differentiation of human bone-marrow derived stem cells and stimulated RAGE expression, ROS generation and apoptosis, while all of these effects were partially attenuated by blocking RAGE (Kume et al., 2005). Moreover, in a murine model of diabetes, the abundance of bone marrow MSCs was found to be significantly decreased compared to non-diabetic mice, while *rage^–^/^–^* mice were protected against those effects and their MSCs had greater differentiation potential (Aikawa et al., 2016). Again, these effects mimic those seen during aging, as BM-MSC differentiation into osteoblasts becomes impaired, osteoblast function is decreased and apoptosis of more mature osteoblasts is increased (Marie, 2014), suggesting that diabetes promotes accelerated aging of bone. Furthermore, BM-MSCs have been reported to show senescence-like changes (Cheng et al., 2011), and the finely balanced fate choice between osteoblasts and marrow adipocytes can be altered by a number of stressors including aging, obesity and diabetes (Chen et al., 2016; Zhuang et al., 2016). Exposure of bone marrow adipocytes to high glucose leads to marked alterations in expression of PPARγ and ADIPOQ, as well as high ROS production (Rharass and Lucas, 2019), while aging leads to increased marrow adipogenesis (Justesen et al., 2001), which presumably contributes to weakening of the bone. Mechanistically, AGE-driven blockade of osteogenesis has been suggested to occur through RAGE-dependent promotion of DNA methylation and inhibition of Wnt signaling (Zhang et al., 2018). Given the wealth of data showing that RAGE influences osteogenesis and osteoclastogenesis, both *in vitro* and *in vivo*, it is surprising that RAGE deletion in animals does not seem to have major effects on bone tissue, especially in the context of diabetes (Hamada et al., 2010). However, all these studies were conducted in young mice, when the assessment of bone fragility associated with diabetes may not be the most relevant. Therefore, studies assessing bone fragility in older rage^–/–^ animals, with or without diabetes, are required. In addition, since RAGE is almost ubiquitously expressed, it is possible that global RAGE knock-out may trigger bone-independent mechanisms that indirectly affect bone cell biology and give different results in vivo than those seen *in vitro*. Therefore, therapeutic strategies specifically targeting RAGE in the bone, such as D6 esRAGE (Takahashi et al., 2013), may be of particular interest.

## Rage Ligands Alter the Bone Cell Microenvironment and Lead to Myeloid Skewing

Hyperglycemia induced by diabetes in mice increases the number of circulating monocytes, the cell type from which osteoclasts are derived, and leads to elevated RAGE expression and serum levels of the RAGE ligand S100A8/A9 (Leclerc et al., 2009; Teissier and Boulanger, 2019). This monocyte proliferation is dependent on S100A8/A9 and RAGE expression in bone marrow cells and is prevented by genetic knock-out or pharmacological inhibition. Moreover, in transplant experiments, WT mice receiving bone marrow from *rage^–^/^–^* mice were protected against hyperglycemia-induced myelopoiesis (Nagareddy et al., 2013; Flynn et al., 2020).

**FIGURE 3:**
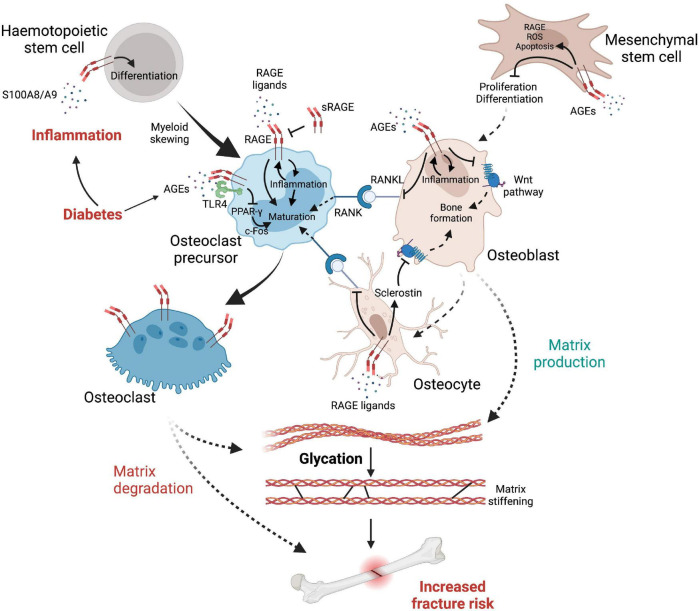
The complex interplay between cells, inflammatory factors, AGEs and RAGE leading to elevated fracture risk in diabetes. Diabetes reduces osteoblast production while promoting myeloid skewing from hematopoietic stem cells, notably *via* RAGE signaling, favoring the production of osteoclast precursors. RAGE initiates a self-sustaining pro-inflammatory response which promotes differentiation of osteoclast precursors into fully mature osteoclasts that express high levels of RAGE. Osteoclast differentiation is further facilitated by RANKL up-regulation, initiating osteoclast maturation signals when interacting with RANK, which is promoted by RAGE signaling from osteocytes and osteoblasts. However (see text for details), AGEs can also negatively affect osteoclast maturation *via* RAGE-independent mechanisms. In contrast, the Wnt pathway is inhibited either directly by RAGE signaling or by its upregulation of sclerostin produced by osteocytes, therefore provoking an imbalance in favor of bone matrix degradation. Glycation of the organic matrix itself leads to structural changes such as matrix stiffening, increasing brittle fracture risks and impairing bone remodeling.

Myeloid skewing is commonly found during aging, as inflammaging favors the production of common myeloid progenitors over that of common lymphoid progenitors (Rossi et al., 2005; Pang et al., 2011; Lee and Yu, 2020), suggesting that inflammatory processes, including RAGE signaling, increase the proliferation of these cell subtypes and therefore may promote the production of osteoclast progenitors (Figure 3). *In vitro*, RAGE expression in osteoclasts increases with differentiation, while osteoclasts derived from *rage/-* mice showed a diminution in proper osteoclast differentiation, actin rings and podosome formation, as well as decreased resorption potential, potentially as a consequence of reduced production of degrading enzymes MMP-9 and cathepsin K (CTSK). Notably, BMMs from *rage/-* mice have a diminished response to vitronectin, a ligand for the integrin α_v_β_3_, important in osteoclastic maturation and function (Zhou et al., 2006). It should be noted that glycation of other glycoproteins, such as fibronectin, greatly perturbs their interactions with integrins, while it allows their interaction with RAGE (Dhar et al., 2017). Hence elevated AGEs in diabetes are likely to disrupt vitronectin/integrin interaction and subsequent osteoclast maturation, which may shift the bone remodeling balance and predispose to bone fragility in diabetes.

## Impact of Advanced Glycation End-Products and Rage on Fracture Risk in Diabetic Bone

Given the major role of AGEs and RAGE in diabetes and inflammatory processes, and the presence of very long-lived proteins which are susceptible to glycation (Sivan et al., 2006), the accumulation of AGEs in bone appears to have a pivotal role in bone deterioration in diabetes (Farlay et al., 2016). Levels of the AGE pentosidine associate strongly with fracture risk (Yamamoto et al., 2008, 2009; Schwartz et al., 2009; Neumann et al., 2014; Tamaki et al., 2018) and moderate or severe vertebral deformity in older adults with type 2 diabetes, but not in those without diabetes (Schwartz et al., 2009). In a cohort of older Japanese diabetic patients, this association was mainly found in post-menopausal women, but not in men (Yamamoto et al., 2008). Lower sRAGE levels and a low sRAGE to pentosidine ratio were associated with increased incidence rate of fractures in men 65 years old or older independently of BMD, suggesting that pentosidine, in association with sRAGE levels, may be a better marker than BMD for fracture susceptibility in older men (Tamaki et al., 2018) and in diabetic patients (Yamamoto et al., 2009). In younger patients with T1D (mean age 45 years) high pentosidine levels, but not sRAGE levels, were associated with prevalent fractures (Neumann et al., 2014). AGEs are not simply markers of fracture risk, but are pathogenic -local injection of the AGE, CML, into mice significantly delayed bone healing, recapitulating the effects of diabetes (Santana et al., 2003). CML-collagen, but not collagen alone, induced apoptosis of bone cells in the skull *in vivo* and of osteoblasts *in vitro*, through p38 and JNK signaling pathways. This effect was abrogated using anti-RAGE antiserum (Alikhani et al., 2007), strongly suggesting that RAGE is central to the loss of bone cells.

## Senescence as a Possible Causative Factor in Bone Fragility

As discussed above, glycation products and RAGE signaling increase bone fracture risk both in normal aging and in diabetes by adversely cross-linking the collagen matrix, skewing bone cell differentiation, and triggering local inflammation through RAGE signaling. In addition, AGEs also appear to have the capacity to trigger cell senescence.

Senescence represents one potential cell fate on exposure to stress, with other alternatives being transient cell cycle arrest or apoptosis. Senescence is a state of permanent withdrawal from proliferation accompanied by many morphological and functional changes. Senescence drivers include DNA damage, either through exposure to genotoxic agents, or endogenous damage signaling resulting from telomere attrition, as well as oxidative damage, proteostatic stress, viral infection (Kohli et al., 2021), and oncogene activation (reviewed McHugh and Gil, 2018). High levels of the cyclin-dependent kinases inhibitors *p16INK4a* (CDKN2A) and *p21Cip1* (CDKN1A), together with elevated p53, ensure the permanence of cell cycle arrest. However, such cells are resistant to apoptosis. Importantly, senescence can also be driven by inflammatory signaling—exposure to IL-6 alone appears to be sufficient (Kojima et al., 2013)—so a pro-inflammatory milieu, such as that resulting from RAGE signaling in diabetic bone, can result in cells entering senescence. Indeed, senescent cells have been described in both ageing and diabetic bone (Farr and Khosla, 2019).

In the short term, senescence is an adaptive response and beneficial in preventing tumor formation as well as aiding in wound healing—indeed, it is a key mechanism in limb regeneration in axolotls (Walters and Yun, 2020) and in early mammalian development (Muñoz-Espín et al., 2013; Storer et al., 2013). It is also vital for the function of the mammalian placenta and in determining pregnancy timing (Cox and Redman, 2017). In acute stress-driven senescence, senescent cells secrete inflammatory cytokines and chemokines that recruit immune cells, particularly macrophages, to promote clearance and wound healing; this also provides immune surveillance to protect against tumorigenesis. However, chronic low-grade stress or gradual telomere loss across the replicative lifespan of cells, combined with gradual diminution of the immune response through immunosenescence, leads to accumulation of senescent cells within tissues (van Deursen, 2014). This is observed in normal aging, and more so in progeroid syndromes with accelerated aging including Hutchinson-Gilford progeria syndrome (HGPS) and Werner syndrome (Cox and Faragher, 2007).

Senescent cells are enlarged (at least *in vitro*), often multinucleate, and show accumulation both of actin stress fibers and granular inclusions that are thought to reflect a failure of autophagy, as well as increased nuclear size with prominent nucleoli and altered heterochromatin patterns, increased mitochondrial mass with high ROS, and dysfunctional lysosomes (Hernandez-Segura et al., 2018). Most notably, while senescent cells are metabolically active, they show marked changes in gene expression and adopt a secretory phenotype, producing a large range of cytokines, chemokines, growth factors and tissue remodeling factors, collectively known as the senescence-associated secretory phenotype, or SASP (Coppe et al., 2008; Basisty et al., 2020). The SASP can drive bystander senescence (also known as secondary senescence) through a paracrine effect (Acosta et al., 2013). While the composition of the SASP varies both by cell type and senescence inducer, several key factors including IL-6, are common to all (Basisty et al., 2020).

Chromatin rearrangements, including a shift in methylation patterns, emergence of senescence-associated heterochromatin foci (SAHFs) (Aird and Zhang, 2013) and formation of new enhancer-promoter loops (Olan et al., 2020) are thought to contribute to the changes in gene expression patterns seen in senescent cells. Moreover, changes to the nuclear scaffold, including weakening and loss of parts of the nuclear lamina, allow accumulation of chromosomal fragments in the cytoplasm (CCFs; Zhang et al., 2007) which trigger innate immune signaling through the cGAS-STING pathway, driving NFκB activation and reinforcing inflammatory cascades (Glück et al., 2017) with direct relevance in the skeleton (Guo et al., 2021), as well as leading to secretion of chromatin proteins HMGB1/2 (Zirkel et al., 2018). Notably, HMBG1 is a ligand for RAGE (Frimat et al., 2019) leading to self-reinforcing inflammatory signaling. The presence of metalloproteases in the SASP also leads to local tissue damage particularly through collagen breakdown, which is likely to have a significant effect in the bone microenvironment, contributing to bone fragility. This is particularly apparent in premature aging Werner syndrome, where patients show highly accelerated accumulation of senescent cells (Faragher et al., 1993, reviewed Cox and Faragher, 2007) and atypical fractures accompanied by loss of trabecular bone (Pignolo et al., 2008). Accelerated senescence of multiple bone cell types in this syndrome, which is driven by failure of DNA helicase/exonuclease WRN and hence prevalent DNA damage, is likely to contribute to the extreme bone fragility through both loss of specialized cell function together with very high SASP levels. Similarly, patients with premature ageing through mutation of CTC1 (conserved telomere maintenance component 1) which leads to genome-wide DNA damage and presumably high senescence burden have elevated risk of atypical bone fracture (Sargolzaeiaval et al., 2018). While a paracrine effect of the SASP has been known for some time, it is becoming apparent that senescent cells may have systemic effects, potentially through the release of SASP-containing endosomes into the blood stream. Indeed, injection of senescent cells into young mice leads to premature onset of a number of age-related pathologies at sites distant from the original senescent cells (Xu et al., 2018). Hence senescent cells are prime candidates for causation of pathologies associated with aging, including bone fragility, and many strategies are being tested either to remove senescent cells or to modify their phenotype and reduce detrimental effects (see “Therapies” below).

In addition to aging, progeroid syndromes, DNA damage, and/or oncogene induction, both obesity and diabetes are associated with an increase in the senescent cell burden. The two are tightly linked: obesity is a major cause of insulin resistance and a key risk factor for pancreatic beta cell failure and T2D development. In obesity, senescent cells accumulate in adipose tissue, liver and brain, while decreasing their burden alleviates adverse related phenotypes including metabolic dysfunction, fatty liver and anxiety (Schafer et al., 2016; Ogrodnik et al., 2019). Thus, adipocyte cell senescence in obesity is a potential contributor to the development of insulin resistance and diabetes.

Senescent cells have been described in both ageing and diabetic bone (Farr and Khosla, 2019), with increased senescence of both osteocytes and myeloid cells observed in old versus young male mice (Farr et al., 2016). Consistently increased senescent cell burden in stromal cell populations was found in aged mice, but surprisingly, removal of these senescent cells by daily administration of the senolytic agent navitoclax for 2 weeks resulted in trabecular bone loss, most probably due to impaired osteoprogenitor function (Sharma et al., 2020). Alveolar bone osteocytes have also been found to display senescence characteristics including increased senescence-associated distension of satellites (SADS), *p16Ink4a* mRNA expression and SASP factors in aged compared to young mice, potentially contributing to age-associated alveolar bone loss (Aquino-Martinez et al., 2021). Importantly, bone cell senescence has also been observed with human ageing, with reports of increased *p16Ink4a*, *p21Cip1* and SASP markers in bone biopsies from older women (mean age 78 years) (Doolittle et al., 2021).

## Diabetes Leads to Increased Cell Senescence

Diabetes is associated with accelerated biological ageing in humans (Bahour et al., 2022). For example, incubation of cells *in vitro* with diabetes-mimicking glucose concentrations stimulates senescence in endothelial cells, renal mesangial cells, preadipocytes, and skin fibroblasts, *via* induction of mitochondrial ROS and oxidative stress (Blazer et al., 2002; Ksiazek et al., 2008; Cramer et al., 2010; Liu et al., 2014). Adaptive changes in the pancreas in T2D, including hyperproliferation to compensate for failures in insulin signaling, may lead to pancreatic cell senescence (Cox, 2008). Furthermore, type 2 diabetes is a highly penetrant feature present in ∼70% of all Werner syndrome patients (Cox and Faragher, 2007), so in addition to DNA damage-driven premature cell senescence, and direct glycation, patients also suffer the added detrimental effect of AGEs triggering RAGE-dependent inflammation, which can drive further senescence. Furthermore, accumulation of senescent pancreatic β cells in non-obese diabetic (NOD) mice and T1D patients has been reported, with increased Bcl-2 expression (i.e., apoptosis-resistance) and secretion of the SASP (Thompson et al., 2019). Importantly, elimination of senescent β cells in NOD mice using BH3-mimetics (which disrupt Bcl-2 protein interactions) overcame the senescence block to apoptosis, mitigated immune-mediated β cell destruction, enhanced insulin secretion and preserved insulin secretory capacity. Thus, removing senescent β cells essentially prevented many phenotypes associated with T1D in NOD mice (Thompson et al., 2019). Though it is still early days for human studies, a 3 day course of treatment of diabetic patients with diabetic kidney disease using the senolytic drug combination D + Q (dasatinib plus quercetin, see “Therapies” below) was found to decrease senescent cell abundance in visceral adipose tissue and reduce serum SASP factors 11 days after completion of treatment (Hickson et al., 2019, 2020), suggesting that senolytic drugs may be powerful new tools in the clinical arsenal against diabetes.

## Senescent Cells Are Pathological in Diabetic Bone

Identifying senescent cells *in vivo* has been challenging because none of the currently available markers of senescence are unique to the senescent state. A widely used marker is SAβgal, a lysosomal form of beta galactosidase active at pH∼6, and hence indicative of dysfunctional lysosomes (Dimri et al., 1995), also marks quiescent cells and those with very active lysosomal function including macrophages and macrophage-like cells such as CNS microglia and bone osteoclasts. Instead, a combination of markers is needed to assess the presence of senescent cells in bone (Farr et al., 2016). For example, senescence-associated distension of satellites (SADS) and TIFS (sites of DNA damage at telomeres marked with 53BP1) are characteristic of senescent cells. Using these markers in mouse models of T2D (high fat diet/streptozotocin-treated animals), the percentage of senescent osteocytes was found to be significantly higher in diabetic animals compared with non-diabetic controls (Eckhardt et al., 2020). This finding was reinforced by the observation of elevated levels of senescence markers p21Cip1 (CDKN1), p16Ink4a (CDKN2) and SASP matrix metalloproteinases *Mmp3, Mmp9, Mmp12, Mmp13* in osteocyte-enriched bone samples derived from the diabetic mice compared to controls, which also correlated with high levels of the AGE carboxymethyllysine (CML) in bone and serum of the diabetic animals (Eckhardt et al., 2020). Such findings are strongly supportive of the idea that diabetes predisposes to high levels of senescence in the bone, and that multiple bone cell types including long-lived terminally differentiated osteocytes, like other post-mitotic cells including neurons (Sapieha and Mallette, 2018; von Zglinicki et al., 2021), are still capable of becoming senescent (reviewed in Farr and Khosla, 2019). This has major implications for bone fragility, since senescence-induced loss of function of the “master-regulator” sensing and signaling roles of the osteocyte is likely to lead to imbalances between bone formation and bone resorption, which in the context of a proinflammatory microenvironment resulting from both AGE-dependent RAGE signaling and the SASP, will predispose toward reduced bone formation and possibly increased bone resorption; digestion of collagen by SASP metalloproteases is also likely to increase fracture risk. Factoring in the contribution of senescent cells to bone fragility both in aging and in diabetes is therefore important in designing therapies that improve not only glucose homeostasis but also minimize bone fragility.

## Therapies to Ameliorate Bone Fragility in Diabetes

### Current Diabetic Therapies

A number of currently available therapies for diabetes, such as DPP4-inhibitors, GLP-1 receptor agonists and SGLT2 inhibitors, mostly have a neutral effect on bone health, except for rosiglitazone, a thiazolidinedione that was shown to decrease bone mineral density and increase fracture risk in post-menopausal women (Bilezikian et al., 2013). Therapies that carry a risk of developing hypoglycemia (a drop in blood sugar below normal values) such as insulin analogs and sulfonylurea (which increase insulin secretion), may be associated with increased risk of falling which in turn may increase the risk of fractures.

Metformin is a first line therapy for most T2D patients. While acute single dosing can dramatically lower blood glucose levels through preventing glucose transport from the intestine (Horakova et al., 2019), therapeutic effects in diabetic patients occur through inhibition of gluconeogenesis in the liver (Madiraju et al., 2014). However, metformin has pleiotropic actions across the body, activating AMP-activated kinase (AMPK) in multiple cell types including osteoblasts, inhibiting mTOR (Kickstein et al., 2010; Nair et al., 2014; Pérez-Revuelta et al., 2014), reducing reactive oxygen species (ROS) (Batandier et al., 2006; Zheng et al., 2012; Bridges et al., 2014), decreasing DNA damage and inflammation (Algire et al., 2012; Saisho, 2015), and activating autophagy (Xie et al., 2011; Song et al., 2015).

Importantly, metformin has been shown to reduce cellular senescence in non-diabetic mice and humans (Jadhav et al., 2013; Moiseeva et al., 2013). Metformin treatment inhibited senescence markers in bone marrow mesenchymal stem cells (BM-MSCs) derived from nephrectomized mice, a model of chronic kidney disease (CKD). The percentage of BM-MSCs positive for senescence markers p16Ink4a, phospho-p53, SA-β-gal (senescence-associated-β-galactosidase), and 53BP1 (p53-binding protein) was lower in metformin-treated compared to control untreated CKD-MSCs, and the SASP was also reduced (Kim et al., 2021). Metformin also stimulates expression of OPG and reduces RANKL expression by osteoblasts *in vitro* (Mai et al., 2011), resulting in decreased osteoclast differentiation.

Notably, metformin inhibits AGE-dependent inflammation in macrophages, at least partly by limiting RAGE signaling (Zhou et al., 2016). It also prevents AGE-induced apoptosis, oxidative stress, expression of RAGE and poor osteoblast differentiation *in vitro* (Schurman et al., 2008). In a rat model of diabetes, metformin prevented diabetes-induced RAGE expression, anti-osteogenesis effects and microarchitecture alterations (Tolosa et al., 2013). Importantly, in humans, trabecular bone score was improved on metformin treatment (Jackuliak et al., 2019). Metformin is to be tested as a general therapy to counter biological aging processes in the large-scale human TAME trial (Targeting Aging with MEtformin), with a mixed endpoint of progression to one of a number of age-related diseases (Barzilai et al., 2016). While bone fracture is not a primary readout of the trial design, it will be interesting to determine whether metformin will lower the risk of bone fracture in the older people in the trial, many of whom are likely to have or develop prediabetes.

### AGE/RAGE Antagonists Reduce Inflammation but Effects on Bone Are Variable

RAGE represents a further potential target for treatment of bone fragility in diabetes. In a murine model of rheumatoid arthritis, administration of an acidic oligopeptide tagged esRAGE (D6esRAGE), which preferentially targets the bone, significantly reduced lesion progression, with significantly fewer synovial lesions, and less cartilage and bone destruction together with a large diminution of TNF-α, IL-1, and IL-6 levels (Takahashi et al., 2013). However, while short term administration of the RAGE antagonist Azeliragon (TTP488) in young and older mice recapitulated some of the effects of RAGE knock-out, such as inhibition of osteoclast differentiation (Ding et al., 2006; Zhou et al., 2006; Hamada et al., 2010), the number of osteoblasts was unexpectedly lowered (Davis et al., 2019). Previous data *in vitro* suggested that RAGE expression limits osteoblast proliferation and osteogenesis (Li et al., 2012; Zhang et al., 2018) while they appear unaffected by RAGE knock-out in young mice (Ding et al., 2006; Hamada et al., 2010). Paradoxically, since RAGE is typically involved in pro-inflammatory events (Teissier and Boulanger, 2019), and inflammatory markers are low in the serum of *rage^–^/^–^* mice (Ding et al., 2006), inhibition of RAGE using Azeliragon appears to increase inflammation, particularly proinflammatory markers IL-6 and monocyte chemoattractant protein-1 (MCP-1) in the tibia of treated mice (Davis et al., 2019). These findings suggest that Azeliragon may have unwanted off-target effects and therefore that more investigation is needed to elucidate the functioning of Azeliragon in bone physiology.

The impact of AGE antagonists on bone health is even less clear-cut. Daily administration of aminoguanidine (AG) or N-phenacylthiazolium bromide (PTB), both reported as anti-AGE molecules, delayed wound healing of the rat mandible. This delay was associated with reduced RAGE and TNF-α expression (Tsai et al., 2015). In contrast, the same drugs slightly accelerated wound healing of soft tissue while also decreasing most inflammatory signals by day 7 post-injury (Chang et al., 2014). These results reinforce the idea that inflammation may serve a positive functional role bone metabolism, especially during wound healing, and that by reducing inflammation, AG and PTB may delay bone wound healing. However, *in vitro*, both AG and pyridoxamine were able to limit glucose-dependent glycation of cortical bone samples and significantly reduce glycation-induced bone deterioration (Abar et al., 2018). Developing therapies based on the AGE-RAGE axis is therefore complicated by the apparently paradoxical effects of drugs compared with genetic knock-out of RAGE and possible tissue-specific effects.

Rather than focussing on RAGE signalling per se, some recent studies have sought to improve bone integrity by limiting the impact of AGEs through other axes. For example, spironolactone, a competitive inhibitor of aldosterone, reversed the deleterious impacts caused by potent glycating agent methylglyoxal, reducing oxidative stress and improving multiple readouts of osteogenesis in an osteoblastic cell line (Park et al., 2021b). Treatment with ALT-711 (also known as alagebrium), described as a potential AGE cross-link breaker, reduced bone porosity and total AGE burden in bone, although it had no overall effect on bone mechanics (Chen et al., 2020). *In vivo*, adrenomedullin 2 was able to circumvent the deleterious effects of AGEs on osteogenesis by increasing PPAR-γ expression, restoring the ultimate load, energy to failure, elasticity modulus and distraction osteogenesis of bones of diabetic rats (Wang et al., 2021). Since AGEs promote inflammatory signalling, and inflammation is associated with reduced bone integrity, approaches to reduce inflammation have been tested for their impact on bone. Direct blockade of IL-1, or indirect downregulation by the use of IL-4, was found to limit bone destruction in a mouse model of arthritis (Joosten et al., 1999a,b; Hernandez et al., 2020); it also appeared beneficial in humans (Dinarello et al., 2012). Anakinra, a small recombinant agonist of the IL-1 receptor, is now licensed for treatment of rheumatoid arthritis (RA). It has modest disease-modifying activity in RA (Mertens and Singh, 2009), and has been reported to lead to positive outcomes in diabetic patients with gout that was refractory to other interventions (Vitale et al., 2015). Hence strategies that aim to remove AGEs or overcome their oxidative and inflammatory effects show some promise of benefit in diabetic bone, but whether cytokine inhibitors (including biological anti-inflammatory therapies) will impact on bone fracture risk in patients as they age is currently unknown.

### Drugs That Kill Senescent Cells Improve Bone Outcomes

As discussed above, a number of cell types in the bone microenvironment undergo senescence during aging and senesce prematurely in patients with diabetes, with senescent myeloid cells and osteocytes developing the SASP (Farr et al., 2016). Hence eliminating senescent cells represents a new approach to disease modification. Beneficial effects on bone structure and strength have been demonstrated using both genetic and pharmacological approaches to remove senescent cells. In transgenic animals with senescence-specific caspase expression (under control of the p16Ink4a promoter), clearance of senescent cells was accompanied by better spine and femur micro-architecture and strength in treated males compared to vehicle-treated mice (Farr et al., 2017). However, such genetic means of senescent cells killing cannot be applied to the patient clinic, so drugs that have senolytic activity (i.e., specifically kill senescent cells) have been sought. Of these, a combination of the FDA-approved tyrosine kinase inhibitor dasatinib (D) and quercetin (Q), a plant flavanol, appears effective in removing senescent cells from many tissues. This D + Q combination is effective even when administered only periodically through a so-called “hit-and-run” mode of action, as shown in the improved vertebral and femoral microstructure of aged male mice administered D + Q intermittently over a period of 4 months. Notably, fewer osteoclasts and more osteoblasts were observed on the endocortical surface following treatment, with a resulting increase in bone mineral deposition (Farr et al., 2017). Moreover, the diminished osteogenic capability of bone marrow mesenchymal stem cells (BM-MSCs) in aged mice can be corrected by treatment with D + Q (Zhou et al., 2021). This senolytic combination of D + Q has also been reported to reduce senescent cells in adipose tissue of diabetic patients (Hickson et al., 2019, 2020), though the effect on bone in these patients has not yet been described.

As well as being effective even with intermittent dosing regimens, these drugs have a very short *in vivo* half-life, with full drug clearance in a matter of hours, minimizing the risk of side-effects. Dasatinib has previously been reported to prevent osteoclastogenesis through inhibiting kinase c-fms without affecting osteoblast markers osteocalcin and P1NP, hence leading to increased trabecular volume and thickness in cancellous bone (Vandyke et al., 2010) in a mechanism that may not be directly related to senescence. A further senolytic that is looking very promising is the plant flavonoid fisetin, which kills senescent cells (Yousefzadeh et al., 2018) and which may also possess anti-inflammatory properties through induction of NRF2 (Rolt and Cox, 2020).

### Treatments to Prevent Senescence Improve Bone Parameters

Multiple rounds of cell proliferation lead to shortening of telomeres at the ends of chromosomes until eventually they become so short that they can no longer be capped by the protective shelterin complex. This uncapping triggers a p53-dependent DNA damage response that leads to replicative senescence, impairing osteoblast function (Wang et al., 2012). Hence preventing telomere loss may provide a novel route to improve aging physiology including bone (Bernardes de Jesus et al., 2012). Pilot studies in transgenic mice expressing telomerase in osteoblasts or in MSCs demonstrated better bone formation *in vivo* (Shi et al., 2002; Yudoh and Nishioka, 2004; Bär et al., 2016), highlighting the importance of replicative senescence in driving bone loss on aging. Telomerase reactivation is, however, contentious as it is also strongly associated with cancer (Akincilar et al., 2016), although mice derived from embryonic stem cells with extremely long telomeres showed better glucose and insulin tolerance and lower cancer risk than controls (Munoz-Lorente et al., 2019), suggesting that transient telomere lengthening strategies may improve health and avoid cancer risk. While transgenic techniques are not yet ready for human use, nutraceutical telomerase reactivators have been developed (Tsoukalas et al., 2019), which appear to reduce fasting glucose levels and slightly increase bone mineral density in the spine (Harley et al., 2013).

### Bisphosphonates Impact Bone Mineralization Directly but Also Affect Cell Senescence

Bone fragility, particularly resulting from age-associated osteoporosis, is commonly treated with bisphosphonates (BPs). This class of compounds acts in the HMG-Co-reductase pathway of cholesterol biosynthesis, preventing farnesol and geranylgeraniol synthesis. *In vitro*, bisphosphonates (especially alendronate), were shown to prevent the harmful effects of AGEs on bone marrow progenitor cells and osteoblast proliferation, differentiation, function and apoptosis (Gangoiti et al., 2008, 2013; Chuguransky et al., 2016) while they also prevented most deleterious impacts of diabetes on bone in a rat model of diabetes (Chuguransky et al., 2016). By blocking lipidation of the small G proteins Rho, Ras, and Rac in osteoclasts, bisphosphonates can interfere with osteoclastogenesis, and also disrupt the actin cytoskeleton in such a way as to prevent proper attachment of mature osteoclasts to the bone resorption surface, which would be expected to favor bone mineralization. However, because of the intimate crosstalk between key bone cells *in vivo*, it has been suggested that loss of osteoclasts on BP treatment may lead to a concomitant decrease in bone formation by osteoblasts (reviewed in Khosla et al., 2020). Contrary to this hypothesis, in a mouse model of premature aging Hutchinson–Gilford progeria syndrome (HGPS), a treatment combination of the bisphosphonate zoledronic acid together with pravastatin (which also acts in the HMG-CoA-reductase pathway in liver) significantly reduced accumulation of AGEs and improved HGPS-induced bone defects (Cubria et al., 2020). In a triple-blinded trial of 60 post-menopausal women, alendronate was reported to improve fasting plasma glucose, HbA1c and insulin levels in prediabetic patients, possibly by affecting carboxy-osteocalcin (Karimi Fard et al., 2019). Moreover, BPs may act to protect against senescence, and they are now being actively researched as potential senolytic or senomodifying agents. For instance, zoledronate has been reported to act as a geroprotector in mesenchymal stem cells, reducing DNA damage even after irradiation (Misra et al., 2016), through the mTOR pathway, a key node of cell signaling that becomes hyperactivated in senescence (reviewed in Walters and Cox, 2018).

### mTOR Inhibition Improves Bone, Potentially Through Modifying Senescent Cell Behavior

Senescent cells show multiple changes to the transcriptome, proteome and secretome compared with the original cells from which they derived. However, a few key nodes within the signaling pathways of senescence are particularly susceptible to drug intervention, suggesting that such nodes are central in either driving or maintaining the senescent state. Most notable of these is the mTOR kinase. Inhibition of mTOR complex 1 (mTORC1) by the drug rapamycin extends lifespan in species ranging from simple yeasts through to mice (reviewed Cox, 2009; Cox and Mattison, 2009); moreover, rapamycin improves multiple health readouts, including ameliorating cognitive decline in mouse models of Alzheimer’s disease (Richardson et al., 2015) and improving cardiac health in dogs (Urfer et al., 2017). mTOR inhibitors show promise in a range of health conditions including age-related diseases and those affecting patients with diabetes (Walters and Cox, 2018). At the cellular level, inhibition of mTOR leads to significant reduction in multiple senescent phenotypes (Walters et al., 2016). Consistent with a fairly global impact on cell senescence, mTOR inhibitors also reduce SASP production (Herranz et al., 2015; Laberge et al., 2015; Rolt et al., 2019).

**FIGURE 4:**
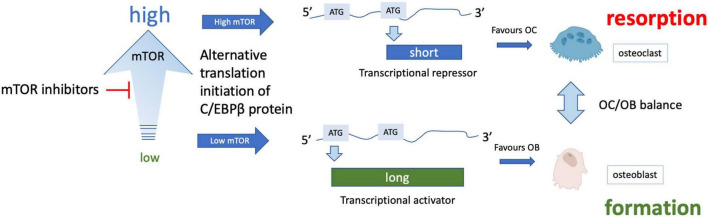
Effect of mTOR inhibition on osteoclast/osteoblast balance. Various isoforms of the transcription factor C/EBPβ can be translated from the same mRNA through translational skipping (also known as leaky ATG scanning) which is regulated by mTOR. Under conditions of high mTORC1 signaling, ATG skipping results in translation of a short isoform that is a transcriptional repressor, while lower mTORC1 signaling allows translation from an ATG toward the 5′ end of the mRNA, forming a longer transcriptional activator form of C-EBPβ (note only 2 isoforms are shown for simplicity). The long isoform is associated with osteoblast differentiation while the short isoform appears to promote osteoclast differentiation (for more details see Smink and Leutz, 2010).

mTOR inhibitors have been shown to have significant benefit in specific cases of bone mineral loss. For example, in women with breast cancer taking aromatase inhibitors, the mTOR inhibitor sirolimus (otherwise known as rapamycin) was found to attenuate bone loss (reviewed Hadji et al., 2013). Similarly, in the BOLERO-2 trial, treatment with the related rapalogue everolimus led to bone improvement in women taking anti-estrogen therapy for hormone-sensitive breast cancer and slowed progression of existing bone lesions or development of new metastases (Gnant et al., 2013). Stimulation of osteoblastic differentiation of human embryonic stem cells has been reported with mTOR blockade (Lee et al., 2010), while rapamycin appears to prevent bone resorption by osteoclasts. mTOR inhibitors reduce protein synthesis by decreasing mTORC1-dependent phosphorylation of S6 kinase and consequently the ribosomal protein S6. In doing so, such drugs can increase protein quality control and overcome major features of proteostatic stress. This effect on protein synthesis is especially interesting but subtle in bone, as rapamycin-dependent inhibition of mTORC1 skews translation of the transcription factor C-EBPβ from a short repressor isoform expressed when mTORC is active, to a longer activator isoform, which changes the balance from osteoclast to osteoblast differentiation (Figure 4). This change in favor of osteoblastogenesis could explain the improvements in bone formation following rapamycin treatment.

While this effect of rapamycin on mTOR’s translational role is beneficial in the case of transcription factor C/EBPβ in adult bone, the anti-anabolic effects of rapamycin, with lower overall rates of protein synthesis, may not always be desirable. Most notably, infusion of 200 ng/μl rapamycin into the limb of young rabbits reduced tibial bone growth plate height compared with contralateral controls in the same animals (Phornphutkul et al., 2009), as well as reducing chondrocyte differentiation (when used at 50 nM) (Phornphutkul et al., 2008). Hence while mTOR inhibition may improve bone health in older adults and those with disease conditions such as diabetes, use of this class of drugs in pediatric patients should be very carefully considered.

Rather than reducing protein synthesis *in toto*, as rapamycin is thought to do, second generation catalytic site mTOR inhibitors appear to provide greater selectivity in terms of which mRNAs are translated, impacting predominantly on mRNAs with oligopyrimidine 5′ tracts. Moreover, the non-competitive allosteric nature of rapamycin’s inhibition of mTORC1 means that the enzyme can become saturated even at low concentrations of the drug. By contrast, the ATP-competitive 2nd generation inhibitors show much tighter dose response curves for cell proliferation (LSC, unpublished data), suggesting that they may provide the option of fine-tuning doses according to patient need. Such drugs are well-tolerated even in frail elderly patients with significant co-morbidities (Mannick et al., 2021), and look promising in *in vitro* models of idiopathic pulmonary fibrosis (IPF) (Woodcock et al., 2019). The observed benefits may accrue from anti-senescence action, since IPF patients also benefit from treatment with senolytic drugs (Justice et al., 2019). Given the central role of mTOR in senescence and inflammation, it will be extremely interesting to assess the clinical effects of mTOR inhibition on age-related bone fragility and in patients with diabetes.

## Conclusion

Improving bone health is a priority for people living with diabetes, to reduce both morbidity and mortality. Here, we have discussed the various drivers of bone fragility in diabetes and in aging, which are now starting to converge as patients with diabetes are living to later ages through advances in glucose control. We identify glycation of components of the bone microenvironment, particularly collagen type 1 cross-linking, inflammatory RAGE signaling and cell senescence as key factors that lead to bone fragility. Finally, we discuss the promise of new therapies that address the underlying biology of bone aging, most notably through removal of senescent cells or modulating mTOR pathways, to improve bone health across the human life course and especially in people living with diabetes.

## Author Contributions

TT, RP, VT, and LC conducted the literature searches. TT, RP, and LC co-wrote the manuscript and revised the drafts. All authors approved the final version.

## Conflict of Interest

The authors declare that the research was conducted in the absence of any commercial or financial relationships that could be construed as a potential conflict of interest.

## Publisher’s Note

All claims expressed in this article are solely those of the authors and do not necessarily represent those of their affiliated organizations, or those of the publisher, the editors and the reviewers. Any product that may be evaluated in this article, or claim that may be made by its manufacturer, is not guaranteed or endorsed by the publisher.
